# “White-line” ptosis repair with complications

**DOI:** 10.5935/0004-2749.2024-0002

**Published:** 2024-04-03

**Authors:** Suzana Matayoshi, Alice Carvalho Gouveia de Almeida, Silvana Artioli Schellini

**Affiliations:** 1 Departamento de Oftalmologia, Faculdade de Medicina, Universidade de São Paulo, São Paulo, SP, Brasil; 2 Departamento de Especialidades Cirúrgicas e Anestésicas, Faculdade de Medicina de Botucatu, Universidade Estadual de São Paulo “Júlio de Mesquita Filho”, Botucatu, SP, Brasil

Dear Editor,

Despite the posterior transconjunctival approach was probably the first technique proposed to correct eyelid ptosis^(1)^, a surgeon must gain familiarity with the surgical anatomy of the everted eyelid to pursue techniques and how to deal with complications. The conjunctival Müllerectomy (CM) technique with or without tarsectomy is the most popular posterior approach technique for ptosis repair. However, the “white-line” technique has gained popularity in the last few years mainly because it can repair the anatomical position of the levator aponeurosis to the tarsal plate and spare tissues. This technique is suitable for correcting mild to severe aponeurotic or congenital ptosis, with good levator function, irrespective of the phenylephrine test results, for patients with a thinned eyelid and a high--arched lid fold and those who are reluctant to have a skin incision and a lid scar^(2)^.

**Figure 1:**
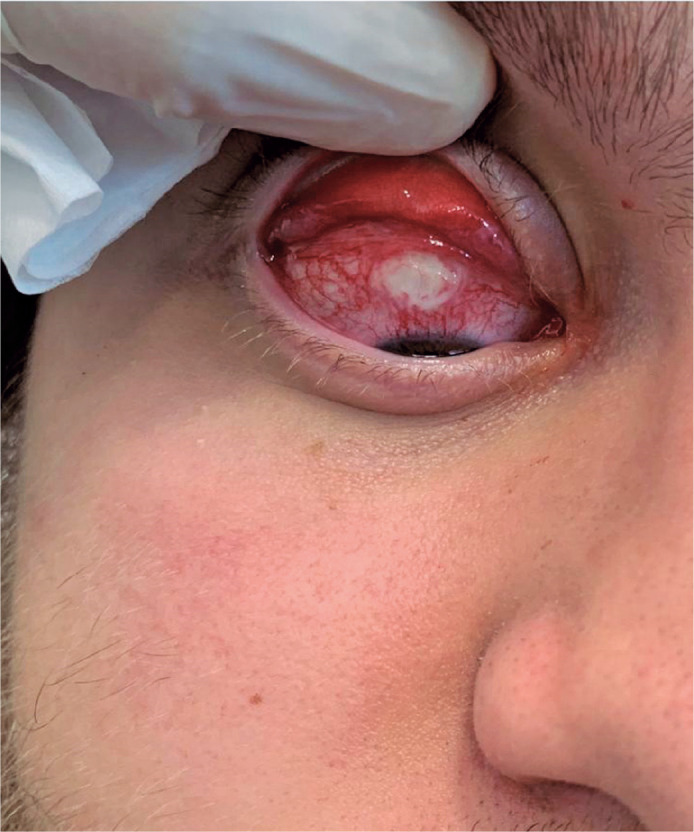
Scleral ulcer in the upper region of the eye, surrounded by dilated conjunctival vessels.

We had an 18-year-old male patient with left-sided Horner’s syndrome secondary to tumor removal in his neck. The patient’s ptosis was previously corrected using the CM technique 1 year ago, with good but transitory ptosis repair. We used the “white-line” technique as usual; however, the elevator complex was reinserted into the tarsal plate using two stitches of 6-0 Prolene thread (Ethicon, Johnson & Johnson, Sao José dos Campos, São Paulo, Brazil). The position and contour of the lid were good immediately after surgery. However, the patient developed left eye pain, and 15 days after surgery, the patient had hyperemic conjunctiva, and a conjunctival and scleral ulcer was observed in the upper sector of the eye (Figure 1). Exploration of the surgical wound revealed a Prolene stitch exposed, touching the sclera. The stitch was removed with good resolution of the condition, and the eyelid maintained a normal position.

Even though the CM technique can be performed using absorbable or nonabsorbable stitches with no increase in the complication rate or reoperation^(3)^, with the “white-line” technique, the levator aponeurosis reinsertion to the Tarsus must be performed using a braided 5-0 absorbable stitch^(1,2)^ because a monofilament thread can lead to rupture of the conjunctiva, exposing the stitch that touches the ocular surface, with the development of scleral/corneal ulcers.It is important to highlight that even with the removal of the suture 15 days after surgery, a good correction of the ptosis remained, showing that the fibrosis between the levator aponeurosis and Tarsus can provide stability and adequate correction of eyelid ptosis.

The advantages of the “white-line” technique are as follows: the maintenance of a good anatomical contour of the upper eyelid, reduced surgical time, and quick recovery. For patients with excessive upper lid skin and laxity, combining this technique with blepharoplasty is possible, preserving the orbital septum intact. Success rates are high at approximately 81.5%-78%^(4)^.Complications, such as a scleral ulcer or corneal abrasion, were not previously reported; however, failures can be result from underor overcorrection, inter-eyelid asymmetry, postoperative hematoma, or infection^(5)^.

In conclusion, the “white-line” technique is an excellent technique for correcting ptosis. Scleral ulcers and corneal abrasion can result from monofilament sutures, which must be avoided.
